# Neurophysiological and Behavioral Responses of Gypsy Moth Larvae to Insect Repellents: DEET, IR3535, and Picaridin

**DOI:** 10.1371/journal.pone.0099924

**Published:** 2014-06-23

**Authors:** Jillian L. Sanford, Sharon A. Barski, Christina M. Seen, Joseph C. Dickens, Vonnie D. C. Shields

**Affiliations:** 1 Biological Sciences Department, Insect Morphology and Physiology Lab, Towson University, Towson, Maryland, United States of America; 2 United States Department of Agriculture, Agricultural Research Service, Henry A. Wallace Beltsville Agricultural Research Center, Invasive Insect Biocontrol and Behavior Laboratory, Beltsville, Maryland, United States of America; AgroParisTech, France

## Abstract

The interactions between insect repellents and the olfactory system have been widely studied, however relatively little is known about the effects of repellents on the gustatory system of insects. In this study, we show that the gustatory receptor neuron (GRN) located in the medial styloconic sensilla on the maxillary palps of gypsy moth larvae, and known to be sensitive to feeding deterrents, also responds to the insect repellents DEET, IR3535, and picaridin. These repellents did not elicit responses in the lateral styloconic sensilla. Moreover, behavioral studies demonstrated that each repellent deterred feeding. This is the first study to show perception of insect repellents by the gustatory system of a lepidopteran larva and suggests that detection of a range of bitter or aversive compounds may be a broadly conserved feature among insects.

## Introduction

The effects of insect repellents have been shown to elicit aversive behavior in numerous insect species through the olfactory system [1], [2]. Three chemicals, in particular, DEET, IR35535, and picaridin, have been shown to be effective insect repellents [3], [4], [5], [6]. A previous study performed on *A. aegypti* demonstrated these three insect repellents elicited responses from a gustatory receptor neuron (GRN) housed within labellar sensilla located at the tip of the proboscis [7]. In this study, we were interested in determining if the same repellents elicited responses from a deterrent-sensitive GRN in a lepidopteran larva. N, N-diethyl-3-m-toluamide (DEET) is a popular insect repellent that is used in commercial bug sprays, including “Repel” and “Off” [8], [9]. DEET is of particular importance because it is capable of repelling numerous insect vectors of harmful diseases. Over the last twenty years, great strides have been made to elucidate the mechanisms of action of insect repellents [1], [2]. While it is known that olfaction plays a major role in mediating the behavioral effects of DEET and other insect repellents, recent research on two selected adult dipteran species clearly demonstrated that repellents may also act through the gustatory system [7], [10]. These authors demonstrated that two adult dipteran species, the vinegar fly *Drosophila melanogaster* [10] and the yellow-fever mosquito *Aedes aegypti* [7] have a gustatory receptor neuron (GRN) housed within the labellar sensilla sensitive to DEET (*D. melanogaster*) and two other insect repellents, IR3535 and picaridin (*A. aegypti*). Lee et al. [10] also showed that DEET deterred feeding in *D. melanogaster*. Additionally, IR3535 and picaridin have been shown to elicit aversive feeding behavior in other mosquito species, such as the black salt marsh mosquitoes, *Ochlerotatus taeniorhynchus* [5]. Another study demonstrated behavioral responses of larvae of the malaria vector mosquito *Anopheles gambiae* to DEET [11]. Nothing is known, however, about the detection of insect repellents by the gustatory sensilla in larvae of other species, such as lepidopteran caterpillars. The gypsy moth larva, *Lymantria dispar*, is a species of horticultural importance as it is notorious for defoliating numerous plant species, particularly forest, fruit, shade and ornamental trees [12], [13].

The chemosensilla of gypsy moth *L. dispar* (Lepidoptera: Erebidae) larvae are located on various sense organs including the antennae, epipharynx, galeae, and maxillary palps [14]. The maxillary palps each bear two pairs of gustatory sensilla, the lateral and medial styloconic sensilla, which have been well characterized [15], [16], [17]. These sensilla in this species and other lepidopteran larvae are each innervated by four GRNs that respond to different taste modalities [15], [16], [17]. A deterrent GRN located in the medial styloconic sensillum is activated by secondary plant compounds such as alkaloids [17] that deter feeding [18], [19].

The goal of our study was to determine if the insect repellents DEET, IR3535, and picaridin are detected by the gypsy moth larval gustatory system. Our behavioral experiments demonstrated that the repellents elicited deterrent feeding behavior when applied to highly favored red oak leaves. Electrophysiological studies revealed that the deterrent GRN housed within the medial styloconic sensillum was sensitive to each repellent and responded to increasing concentrations of these repellents in a dose-dependent manner. Stimulations of lateral sensilla with these insect repellents, did not elicit responses from GRNs. This finding is consistent with a previous study, demonstrating that a deterrent-sensitive GRN is located only in the medial styloconic sensillum [17]. Our results provide evidence that the gustatory system of lepidopteran larvae contributes to the detection of insect repellents and points to the potential usefulness of repellents in the management of immature stages of horticulturally destructive insects.

## Results

### Effects of repellents on feeding

**Figure 1:**
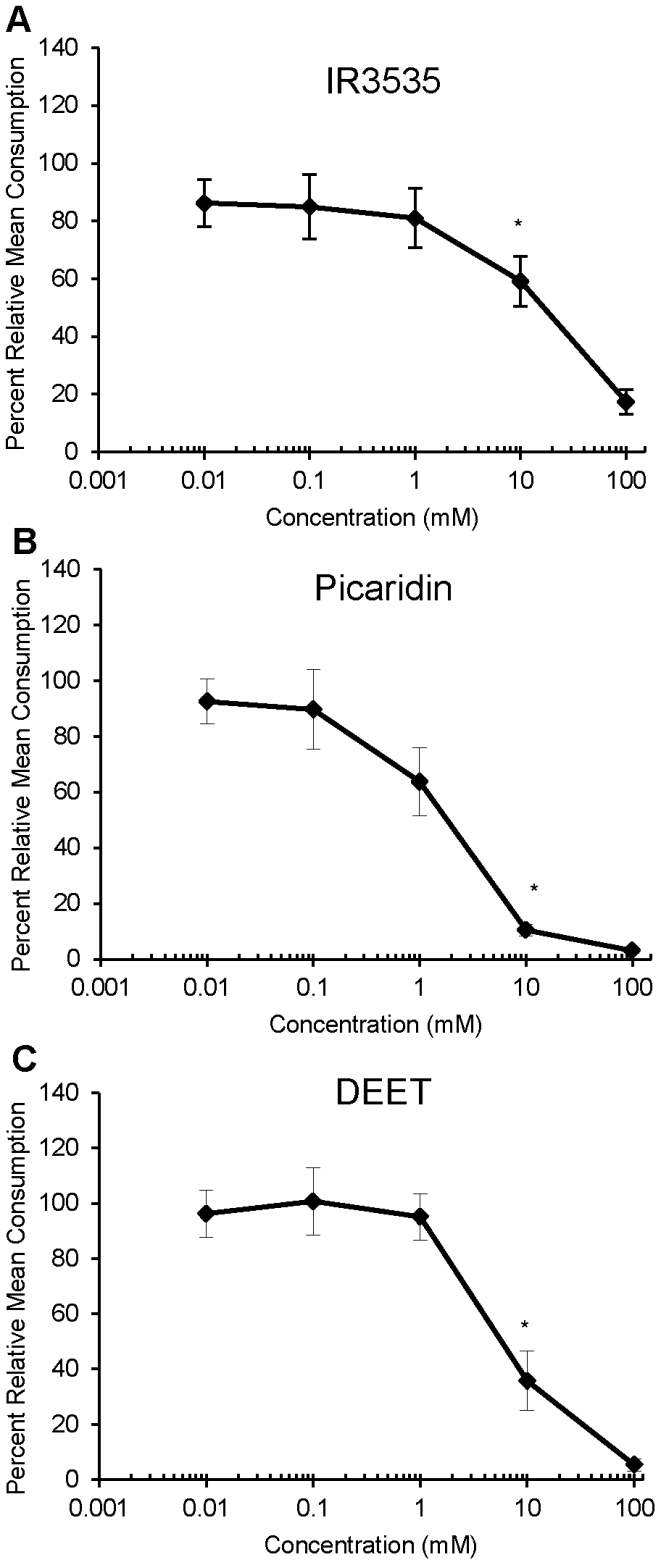
Dose response curves (in percent mean consumption) to increasing concentrations of (A) IR3535, (B) picaridin, and (C) DEET. Asterisks denote the concentration of repellent that significantly decreased feeding relative to the control. Vertical bars represent standard errors.

A dual-choice feeding bioassay [19] tested the effects of the three repellents on feeding by gypsy moth larvae, *L. dispar*. Dose-response curves revealed that all three repellents inhibited feeding and that feeding deterrence increased with increasing concentration of the repellent (Fig. 1.A–C). All three repellents elicited similar dose response curves with a deterrence threshold (DT) value for all three repellents at 10 mM (IR3535, P = 0.03, α<0.05, n = 8–14 sensilla; picaridin, P = 0.02, α<0.05, n = 12–15 sensilla; DEET, P = 0.01, α<0.05, n = 7–10 sensilla). Picaridin and DEET elicited 96.9% and 94.6% feeding deterrence, respectively, at 100 mM (Fig. 1.B and C), whereas the level of feeding deterrence for IR3535 was slightly lower at 82.6% for this concentration (Fig. 1A).

### Effects of repellents on deterrent-sensitive GRN

**Figure 2:**
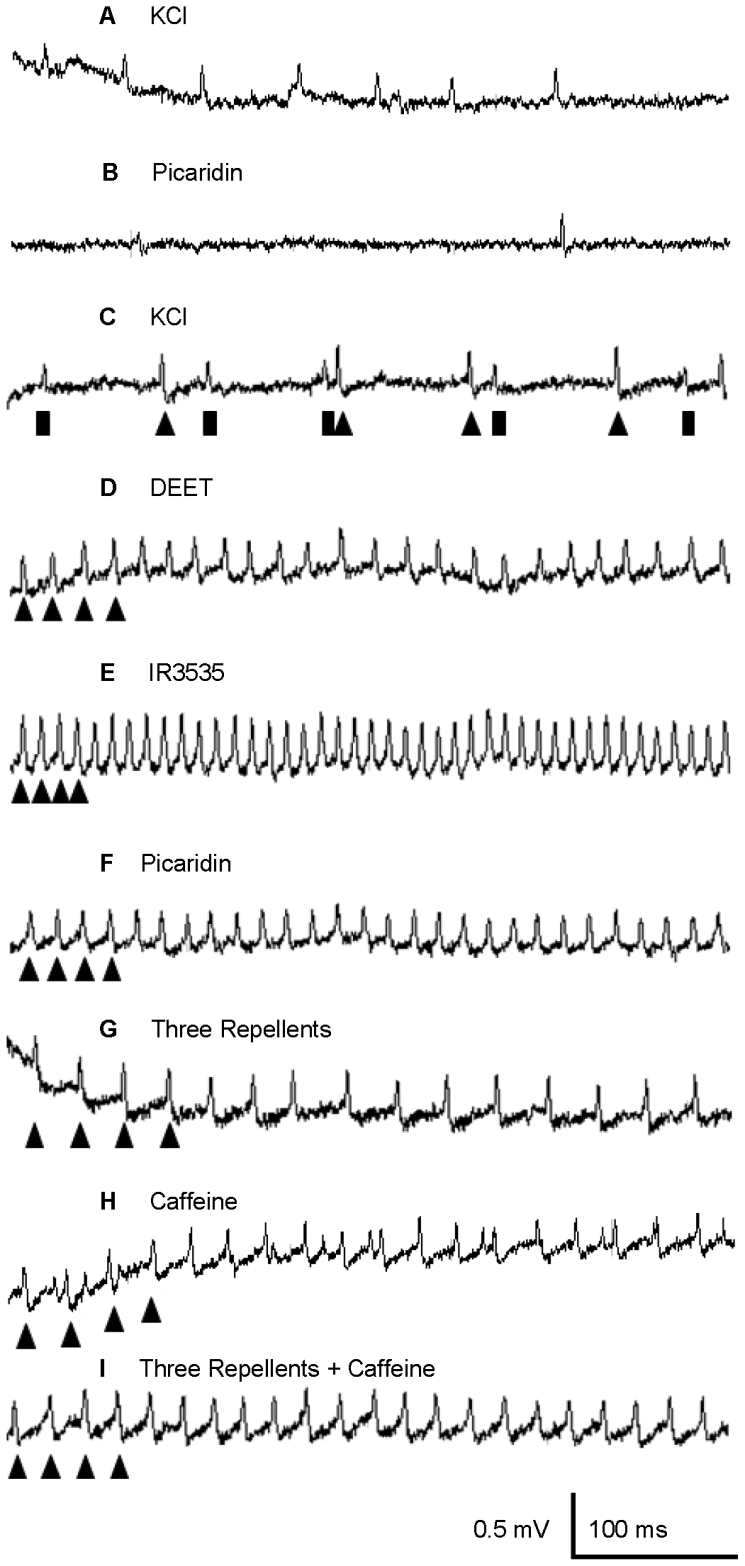
Representative traces of responses elicited from gustatory receptor neurons contained in the medial and lateral styloconic sensilla of *Lymantria dispar* larvae to various stimuli. (A) 30 mM KCl (lateral), (B) 10 mM Picaridin (lateral), (C) 30 mM KCl (medial), (D) 10 mM DEET (Medial), (E) 10 mM IR3535 (Medial), (F) 10 mM Picaridin (Medial) (G) a mixture of 10 mM DEET, IR3535, and picaridin, (H) 10 mM caffeine, and (I) a mixture of 10 mM caffeine and 10 mM each of DEET, IR3535, and picaridin. Stimulations of lateral styloconic sensilla with the three repellents, picaridin (shown above in B), IR3535, or DEET did not elicit responses from the gustatory receptor neurons. Up-arrowheads represent the response of the large-amplitude deterrent-sensitive neuron and bars represent the response of the small-amplitude KCl-sensitive neuron.

**Figure 3:**
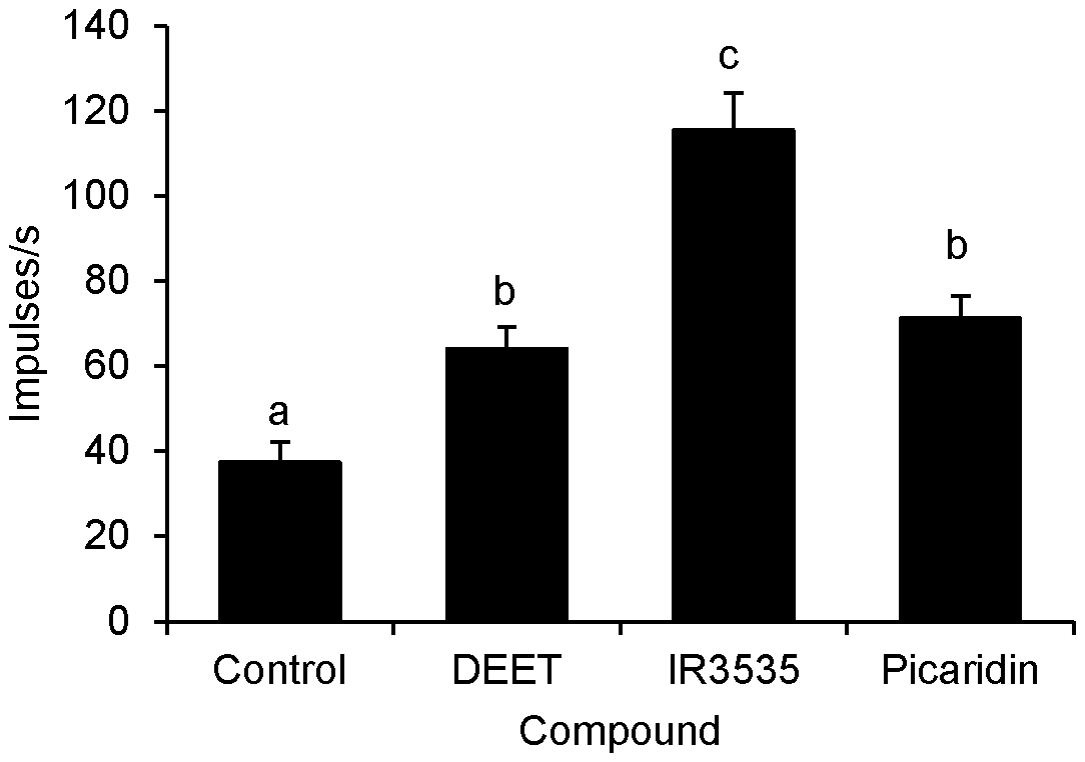
Total number of impulses per second produced in the interval 0.05–1.05 s after initial stimulation of the medial styloconic sensilla in *L. dispar* by the deterrent-sensitive neuron to 10 mM DEET, IR3535, and picaridin compared with the control (30 mM KCl in 10% ethanol). Different letters represent significant differences between groups. Vertical bars represent standard errors.

A single neuron within the medial styloconic sensillum on the maxillary palps fired in response to DEET, IR3535, and picaridin presented either singly (Fig. 2. D–F), or as a mixture (Fig. 2.G). Lateral styloconic sensilla did not respond to these repellents (Fig. 2.B). All three repellents elicited large amplitude spikes, similar in height to the larger of the two spikes elicited by 30 mM KCl in medial styloconic sensilla (Fig. 2.C). Thirty mM KCl elicited only a single large amplitude spike in lateral styloconic sensilla (Fig. 2.A). Previous electrophysiological recordings characterized the neuron with the large amplitude spike as the “deterrent-sensitive cell” in medial styloconic sensilla [17], responding to naturally occurring deterrent compounds, such as caffeine (Fig. 2.H), nicotine, strychnine, and aristolochic acid. To further confirm that the repellents activated the same deterrent-sensitive GRN, neurons within this sensillum were stimulated with a mixture of 10 mM each of the repellents and caffeine. Again, only a single neuron appeared to respond to the mixture (Fig. 2.I). Comparison of the total numbers of spikes produced 0.05–1.05 s after stimulation with each of the repellents with that of the control solution (i.e., 30 mM KCl) revealed that each repellent compound elicited significantly more spikes compared with that of the control (Fig. 3) (repeated measures ANOVA; post-hoc Tukey-Kramer analysis; α<0.05; n = 13–19 sensilla). Among the repellents, IR3535 elicited significantly more spikes (107.3 spikes/s) than either DEET (60.8 spikes/s) or picaridin (67.6 spikes/s) at the 10 mM concentration (Fig. 2.G) (repeated measures ANOVA; post-hoc Tukey-Kramer analysis; α<0.05; n = 13–19 sensilla).

**Figure 4:**
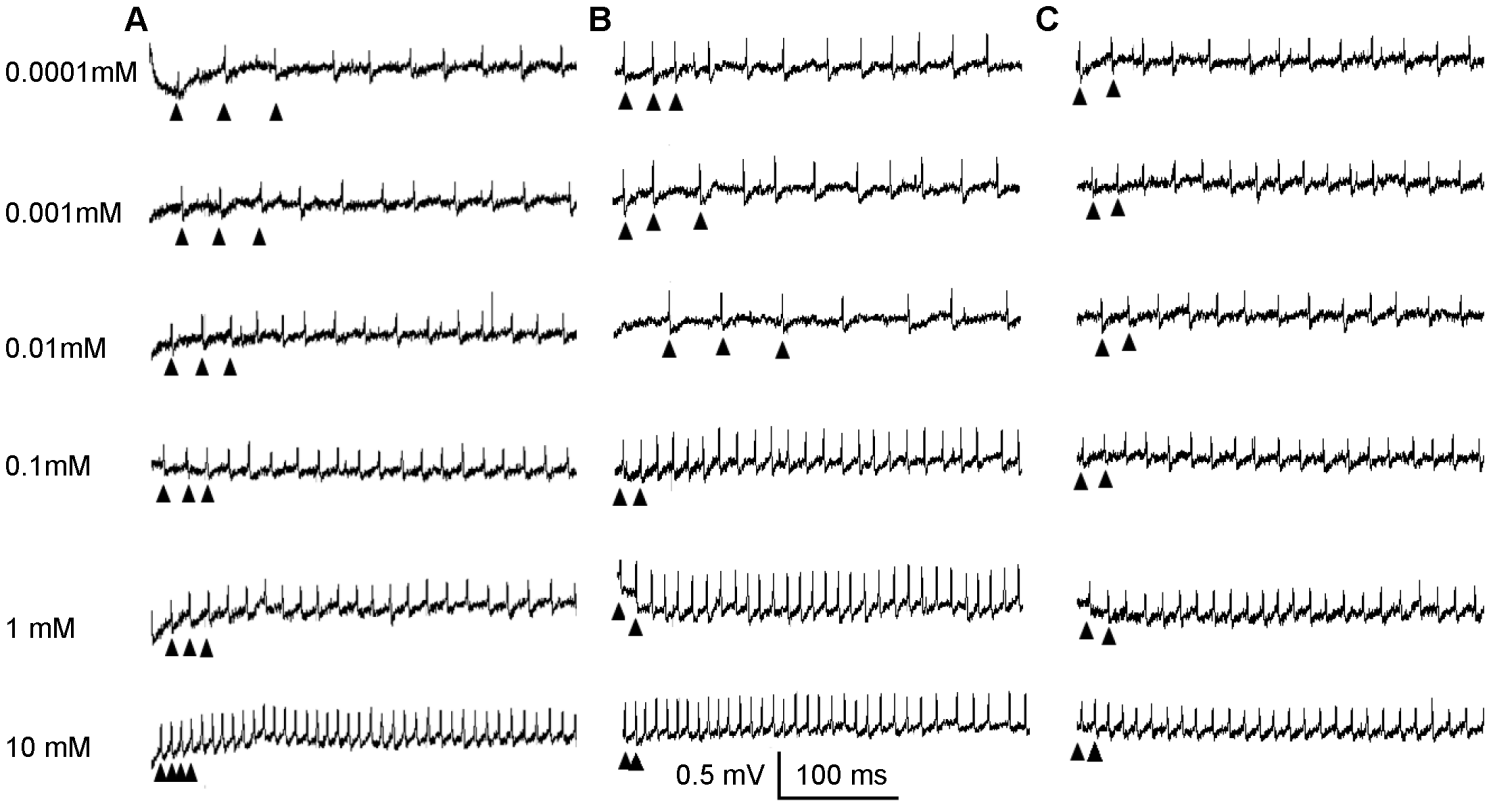
A. Representative traces of responses elicited by the deterrent-sensitive neuron in the medial styloconic sensilla to increasing concentrations of (A) IR3535 (B) Picaridin and (C) DEET. Up-arrowheads represent the response of the large-amplitude deterrent-sensitive neuron.

**Figure 5:**
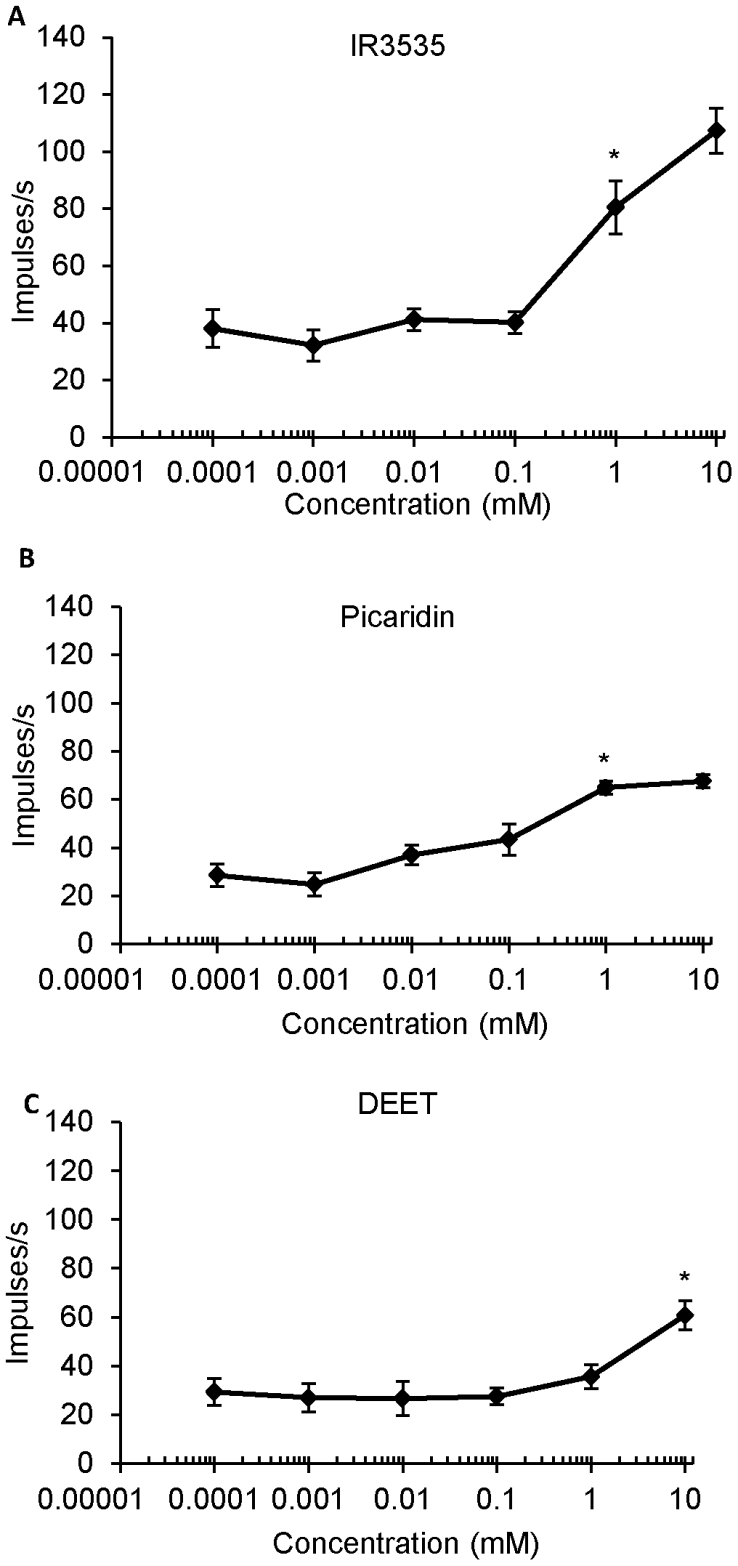
Dose-response curves constructed from numbers of impulses per second in the interval 0.05–1.05 s after initial stimulation by the deterrent-sensitive neuron elicited by increasing concentrations of (A) IR3535, (B) picaridin, and (C) DEET. An asterisk indicates the concentration at which a significant increase in the number of impulses occurred.

Exposure of medial styloconic sensilla to gradually increasing concentrations of the three repellents revealed that the deterrent-sensitive neuron responds in a dose-dependent manner to DEET, IR3535, and picaridin (Figs. 4.A–C). Dose response curves revealed threshold responses to IR3535 and picaridin at 1 mM, while the threshold for DEET was reached only at 10 mM (Fig. 5.A–C) (repeated measures ANOVA; post-hoc Tukey-Kramer analysis; α<0.05; n = 5–6 sensilla). Higher concentrations of DEET were not tested because of the inability of this compound to solubilize in the control solution.

**Figure 6:**
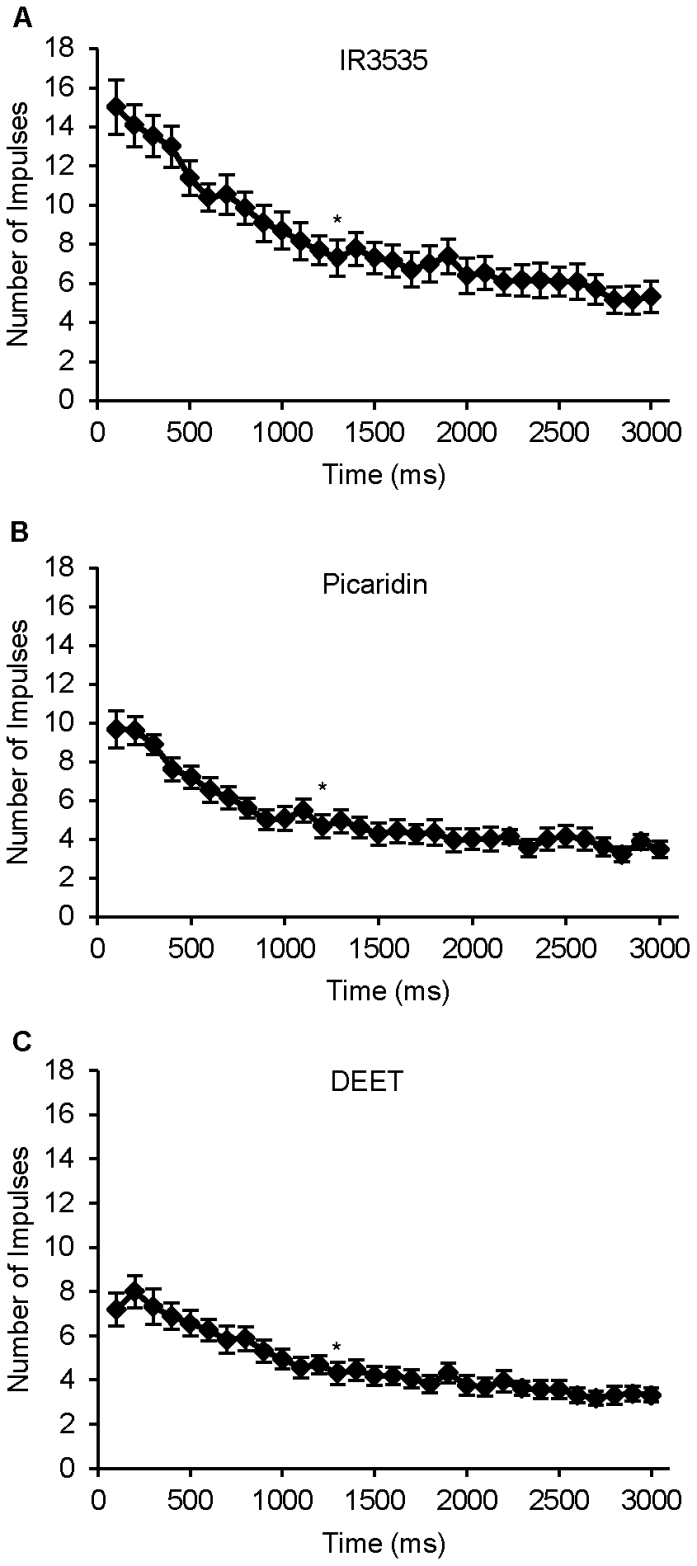
Temporal dynamics of the response of the deterrent-sensitive neuron to 10 mM concentrations of (A) IR3535, (B) picaridin, and (C) DEET. All repellents elicited a phasic-tonic firing pattern which peaked within the first 100–200 ms following stimulus onset and gradually decreased over the next 800 ms for picaridin and 1000 ms for both DEET and IR3535. A tonic rate of activity (marked by an asterisk) occurred after 1200 ms for picaridin and 1300 ms for DEET and IR3535.

All three repellent chemicals elicited a phasic-tonic response from the deterrent-sensitive neuron (Fig. 4). IR3535 elicited the highest firing frequency (15±1.4 spikes) within the first 100 ms after stimulation, while picaridin (9.7±.9 spikes) and DEET (7.2±.7 spikes) elicited fewer spikes within the same time period (one-way ANOVA; post-hoc Tukey-Kramer analysis; α<0.05; n = 13–16 sensilla). The deterrent-sensitive GRN displayed a similar tonic pattern of firing to DEET, IR3535 (1300 ms after initial stimulation) (Fig. 6.A and C), and picaridin (1200 ms after initial stimulation) (Fig. 6.B) (repeated measures ANOVA; post-hoc Tukey-Kramer analysis; α<0.05; n = 13–16 sensilla).

## Discussion

Although the interactions between insect repellents and the olfactory system have been widely studied (see reviews in insect olfaction) [1], [2], relatively little is known about their interactions with the gustatory system. While recent research has demonstrated a direct link between insect repellents and the adult gustatory system [7], [10], nothing was known about how these repellents interact with the larval taste system. Here we demonstrate that insect repellents are detected by a specific GRN in the larva of the *L. dispar* and the presence of the repellents deters feeding.

### Feeding inhibition by repellents

All three repellents inhibited feeding by gypsy moth larvae and feeding deterrence increased with increasing concentrations of the repellent. While all of the repellents were detected at similar concentrations (10 mM), picaridin and DEET elicited 96.9%, and 94.6% feeding deterrence, respectively, at 100 mM; IR3535 elicited 82.6% feeding deterrence at this concentration. Feeding inhibition by the three repellents was similar to that observed for some alkaloids, e.g., sparteine and acridine [17], [18]. Previous work performed with dipteran species have shown that DEET, IR3535, and picaridin elicit feeding deterrent responses in *D. melanogaster* and some mosquito species [5], [10], [20], [21]. In agreement with these studies, we have also found that these repellents elicit feeding deterrence in *L. dispar*.

Our electrophysiological results demonstrated that IR3535 and picaridin elicited robust responses in the deterrent GRN and produced the same DT value (i.e., 1 mM). Similarly, our behavioral results demonstrated that both of these repellents elicited the same DT value (i.e., 10 mM). While, our electrophysiological results showed that DEET elicited the least robust response from the deterrent GRN and had the highest DT value (i.e., 10 mM), our behavioral data demonstrated that the DT value was the same as IR3535 and picaridin (i.e., 10 mM). This could potentially be explained by the fact that DEET may have a more repellent effect on the larval olfactory system, thus the results of the feeding bioassays cannot be explained by gustatory input alone. An olfactory component, which may not be the same for the three repellents, cannot be ruled out.

### Activation of the deterrent GRN by repellents

GRNs of polyphagous caterpillars are capable of detecting multiple chemical classes [22], [23]. One class of chemicals, feeding deterrents, activate a specialized deterrent-sensitive neuron housed within gustatory sensilla in many Lepidoptera [24], [25], [26], [27], [28] and the deterrent-sensitive neuron in larval *L. dispar* responds to the alkaloids, aristolochic acid, caffeine, nicotine, and salicin [17]. Here we show that DEET, IR3535, and picaridin activate the same deterrent-sensitive neuron in *L. dispar*. Stimulation with a mixture of the known feeding deterrent, caffeine, and the three repellents, revealed activity of a single neuron, consistent with selective activation of the deterrent-sensitive neuron. These results are similar to findings in the yellow-fever mosquito *A. aegypti*, where a single GRN was activated by mixtures of the repellent DEET and a feeding deterrent [7].

We found that IR3535 elicited significantly more spikes than either DEET or picaridin from the deterrent-sensitive gustatory neuron in *L. dispar*. Our behavioral data showed that all three insect repellents had the same detection threshold (10 mM). This apparent lack of correlation between electrophysiological and behavioral responses suggest that input from other sensilla may play a role during feeding to regulate CNS output.

Dose response curves to the three repellents showed a threshold concentration of 1 mM for IR3535 and picaridin, while a threshold of 10 mM was found for DEET. The phasic-tonic pattern of neuronal activity elicited by the repellents is similar to the pattern of activity elicited by the alkaloids caffeine, nicotine, and strychnine [17] and may represent activation of the same excitatory transduction pathway by the deterrent-sensitive GRN [17], [29]. It is interesting to note the phasic firing response of the deterrent GRN became tonic at nearly identical times for DEET, IR3535, and picaridin (1300 ms, 1300 ms, and 1200 ms, respectively), which could suggest that all three repellents act in a similar manner on the insect peripheral nervous system.

### Chemical basis for deterrence caused by repellents

DEET, IR3535, and picaridin share an amide moiety, the putative group responsible for mediating repellency in DEET and picaridin [30]. Plants may produce amides as a defense against herbivorous insects as these compounds increase mortality, deter feeding and cause decreased pupal weights [31], [32]. The fact that picaridin elicited a more robust response than DEET in the deterrent sensitive neuron may be explained by the presence of a piperidine moiety in picaridin. Natarajan et al. [30] suggested that the presence of the piperidine ring positioned the amide moiety in a configuration that contributed to its repellent effect. The piperidine moiety found in some alkaloids contributes to feeding deterrence in some Lepidoptera [33], [34]. The increased firing of the deterrent-sensitive neuron elicited by IR3535 compared with DEET or picaridin may be attributed to the presence of an additional ester moiety absent in DEET or picaridin. Interestingly, both caffeine and strychnine contain an amide moiety and both compounds elicit feeding deterrence and activate the deterrent sensitive neuron in *L. dispar* larvae [17], [19].

The results of our study show that the larval gustatory system of *L. dispar* is sensitive to the insect repellents DEET, IR3535, and picaridin. These compounds elicit action potentials from a GRN sensitive to feeding deterrents located in the medial styloconic sensilla of this species and elicit aversive feeding behavior. These GRNs may express conserved gustatory receptor (GR) genes that are required for detection of a broad range of bitter or aversive compounds, a feature of GRNs in Diptera [10], [35], [36]. Moreover, our results suggest repellents as candidate chemicals for use in the horticultural arena as antifeedants to decrease economic losses due to feeding by insect pests.

## Materials and Methods

### Insects

*Lymantria dispar* eggs (New Jersey strain) were obtained from USDA, APHIS, Otis Air National Guard Base in Falmouth, Massachusetts, USA. Caterpillars were reared on a high wheat germ-based artificial diet (Bio-Serv, Frenchtown, New Jersey; MP Biomedicals, Solon, Ohio, USA) and maintained at 27°C±2°C and 60% relative humidity in a 12-h light/12-h dark photoperiod regimen. Fifth instar larvae, 12–18 h postmolt, and 24-h food-deprived were used for both behavioral and electrophysiological experiments. The larvae were naïve to the test compounds prior to experimentation.

### Experimental Chemicals

Experimental chemicals tested, *purity*, and source were *N*, *N*-diethyl-3-methylbenzamide (DEET), *97%*, Sigma-Aldrich, St. Louis, Mo, USA; 1-piperidinecarboyxlic Acid, 2-(2-hydroxytehyl)-, 1methylpropylester (picaridin), *>98%*, LANXESS, Pittsburgh, PA, USA; 3-[N-n-butyl-N-acetyl] aminopropionic acid ethylester (IR3535), Merck KGaA, *>95%*, Darmstadt, Germany. The repellents were dissolved in a 10% ethanol∶30 mM KCl (Fisher Scientific; Fair Lawn, New Jersey) solution to give a final concentration of 10 mM (repellent stock solution). At this concentration, ethanol had no discernible effect on the electrical activity of the GRNs [17]. The majority of tests were carried out with dilutions of this repellent stock solution, in which ethanol comprised 0.1% or less. Both control and solutions of experimental compounds were tested at room temperature (20°C±2°C). Test solutions were given in a randomized order to prevent bias, except for dose response experiments in which solutions were given in order of increasing concentration. At least 3 min were allowed between successive stimulations. All recordings were made between 0900 and 1700 h during light of the photoperiod.

### Behavioral Assay

We used a two-choice feeding bioassay [18] to evaluate the effects of the repellents, DEET, IR3535, and picaridin on the feeding responses of *L. dispar* larvae. Disks (9-mm diameter) were cut from red oak (*Quercus rubra*) (L.) leaves, a plant species highly favored by *L. dispar* larvae [18], [19]. Branches were collected consistently from the same trees between 9–11 a.m. daily (June–August 2012 and 2013) and immediately placed in water and were removed just prior to testing. This was to prevent dehydration of the leaves. Six leaf disks were arranged equidistant (approximately. 4 cm apart) in a circular array in a Petri dish (100-mm diameter, 15-mm depth) (Fisher Scientific, Pittsburgh, PA), alternating control and treatment disks. Disks were held in place 5 mm above the surface by metal pins that pushed through the center of each disk into two pieces of dental wax (Electron Microscopy Sciences, Hatfield, PA). The bottom of the dishes was lined with moistened (2 ml aliquot distilled water) filter paper (90-mm^2^ circle, Grade 1, Whatman, Inc.) to reduce desiccation of the disks.

Feeding experiments were carried out at 24°C (±1°C) until ca. 50% of the total area of either control or test disks had been consumed. Ethanol (Fisher Scientific, Pittsburgh, PA) was used to dissolve each test compound, which was applied at five different concentrations from 0.01 mM to 100 mM. A 20-µl aliquot containing solvent or solvent plus test compound was applied to control and test disks, respectively, to ensure the leaf were completely saturated by the solution. Before placing larvae within the Petri dish, all of the solvent was allowed to evaporate from the leaf surface. At the beginning of the experiment, each larva was placed in the center of a Petri dish. A set of control disks (control disks II), held in the absence of larvae, were retained for comparison purposes for each experiment. Following each experiment, the control, test, and control II disks were oven-dried at 80°C for 48 h. Mean leaf consumption (mg) was determined by subtracting the remaining mass of a test or control disk from the mass of a control disk II. The disks were weighed (Sartorius BP 211 D) (±0.01 mg) and the values were reported as percent relative mean consumption of control consumption.

### Electrophysiology

Electrical responses from gustatory receptor neurons (GRNs) within the medial styloconic sensilla were recorded using an extra-cellular tip-recording method [37], [38]. In brief, the head of a caterpillar was excised then mounted onto a capillary electrode filled with 30 mM potassium chloride. This preparation remained responsive for, on average, 1–2 hours. A silver wire inserted into the capillary served as the indifferent electrode. The recording/stimulating electrode containing a stimulating solution (i.e., repellent) was placed over the tip of a sensillum. The electrode was connected to the input of the preamplifier with a gain of 10× (Syntech Taste Probe, Hilversum, The Netherlands). The electrical activity recorded from neurons within the sensillum was amplified and passed through a bandpass filter set at 100 Hz–1,200 Hz (Syntech, Hilversum, the Netherlands). Recordings were digitized by a 16-bit analog-to-digital interface (IDAC-4 Syntech) and analyzed off-line with Autospike software (version 3.8) (Syntech). For each electrophysiological recording made, each sensillum was stimulated for a total duration of 3 s. Action potentials generated 50 ms after contact of the sensillum by the recording/stimulating electrode were analyzed. For dose response experiments, six different concentrations (0.0001–10 mM) of each of the repellent compounds were tested.

### Data Analysis

For behavioral experiments, a one-way ANOVA followed by a post-hoc Kruskal-Wallis test [39] was run for each of the experimental compounds. The difference between control consumption (100%) and relative mean consumption of disks was tested for each concentration using a paired Wilcoxon signed rank test [39]. A Bonferroni correction for individual comparisons (significance levels of 0.05/5 = 0.01) was used to maintain the experiment-wide error rate of 0.05 [39]. The data were analyzed using Statmost (Dataxiom Software Inc., Los Angeles, CA) and Number Cruncher Statistical System (NCSS) (Kaysville, Utah).

For electrophysiological experiments examining the activation of the deterrent GRN by the repellents, a repeated measures ANOVA (α = 0.05) was used [39]. The repellent solution was the fixed variable and the firing frequency was the response variable. Dose response curves were constructed using the total number of spikes produced by the deterrent-sensitive neuron in the interval 0.05–1.05 s after initial stimulation with increasing concentrations of each repellent. These results were analyzed for statistical differences using a repeated measure analysis of variance (ANOVA) (α = 0.05). A Tukey-Kramer multiple comparison test was used to compare responses of the deterrent GRN to various concentrations of each repellent [39]. The temporal dynamics of responses of the deterrent-sensitive neuron was characterized by the average number of spikes in successive 100 ms time bins across the first 3 s after stimulus onset. A repeated measure analysis of variance (ANOVA) (α = 0.05) was used to compare responses to the different repellents [39]. A Tukey-Kramer multiple comparison test was used to compare responses of the deterrent GRN to determine when a transient phasic pattern of firing changed to that of a sustained tonic pattern of firing [39]. Data was analyzed using Excel (Microsoft Corp. Redmond, WA, USA) and NCSS (Kaysville, UT, USA).
